# Prospective REALITI-A Study: 2-Year Real-World Benefits of Mepolizumab in Severe Asthma

**DOI:** 10.1016/j.chpulm.2024.100107

**Published:** 2024-09-16

**Authors:** Cristiano Caruso, G. Walter Canonica, Manish Patel, Andrew Smith, Mark C. Liu, Rafael Alfonso-Cristancho, Robert G. Price, Rupert W. Jakes, Lydia Demetriou, Antonio Valero, Thomas C. Köhler, Charles Pilette, Geoffrey Chupp, Guy Brusselle, Peter Howarth

**Affiliations:** a UOSD DH Internal Medicine and Gastroenterology, Fondazione Policlinico A. Gemelli IRCCS, Università Cattolica del Sacro Cuore, Rome, Italy; b Department of Biomedical Sciences, Humanitas University, Milan, Italy; c Personalized Medicine, Asthma and Allergy Clinic, IRCCS Humanitas Research Hospital, Milan, Italy; d Department of Respiratory Medicine, University Hospital Wishaw, North Lanarkshire, Scotland; e Divisions of Allergy and Clinical Immunology, Pulmonary and Critical Care Medicine, Johns Hopkins Asthma and Allergy Center, Baltimore, MD; f Value Evidence & Outcomes, GSK, Collegeville, PA; g Biostatistics, GSK, Stevenage, Hertfordshire, England; h Epidemiology, GSK, London, England; i Value, Evidence & Outcomes, GSK, Stevenage, Hertfordshire, England; j Allergy Department, Hospital Clinic Barcelona, CIBERE and IDIBAPS, Barcelona, Spain; k Department of Pneumology, Medical Center - University of Freiburg, Freiburg, Germany; l Department of Pulmonary Medicine, Cliniques Universitaires Saint-Luc, Brussels, Belgium; m Pole of Pneumology, ENT and Dermatology, Institute of Experimental and Clinical Research, UCLouvain, Brussels, Belgium; n Division of Pulmonary, Critical Care, and Sleep Medicine, Department of Internal Medicine, Yale School of Medicine, New Haven, CT; o Department of Respiratory Medicine, Ghent University Hospital, Ghent, Belgium; p Global Medical Affairs, Specialty Medicine, GSK, Brentford, England

**Keywords:** asthma exacerbations, long-term, mepolizumab, oral corticosteroids, real world, severe asthma

## Abstract

**Background:**

Mepolizumab, a monoclonal antibody targeting IL-5, is of proven clinical benefit in severe asthma; however, prospective, long-term, real-world data in severe asthma are required.

**Research Question:**

What is the real-world benefit of 2 years of mepolizumab treatment in severe asthma?

**Study Design and Methods:**

REALITI-A was a 2-year, international, prospective study enrolling adults with asthma on newly initiated mepolizumab 100 mg subcutaneously (physician decision). Outcomes in the 1-year premepolizumab vs 2-year follow-up periods included rates of clinically significant asthma exacerbations (CSEs) (deterioration requiring systemic corticosteroids and/or emergency department [ED] visit/hospitalization), exacerbations requiring ED visit/hospitalization, exacerbations requiring hospitalization, proportion of patients with no exacerbations, median daily maintenance oral corticosteroids (mOCSs) dose, proportion of patients discontinuing mOCSs completely, Asthma Control Questionnaire-5 score, FEV_1_, and adverse events (AEs).

**Results:**

After 2 years’ follow-up, 73% of patients (599 of 822) had no record of mepolizumab discontinuation. During the 2-year follow-up vs premepolizumab period (N = 822), rates of CSEs, exacerbations requiring ED visit/hospitalization, or hospitalization only were reduced by 74%, 79%, and 73%, respectively (odds ratio for no CSEs, 10.0; 95% CI, 7.55- 13.25). Median daily mOCS dose decreased from 10.0 (quartile 1, 5.0; quartile 3, 14.7) mg at week 0 (n = 297) to 0.0 (quartile 1, 0.0; quartile 3, 5.0) mg at weeks 101 to 104 (n = 168), and the proportion of patients discontinuing mOCS increased progressively to 43% at 1 year and 57% at 2 years. There was a 1.53-point reduction in Asthma Control Questionnaire-5 scores from baseline at 2 years. At months 21 to 24, least square mean FEV_1_ improved by 142 mL from baseline. Ninety (11%) and 7 (< 1%) patients experienced mepolizumab-related AEs and serious AEs during the follow-up period, respectively.

**Interpretation:**

In patients with severe asthma, real-world mepolizumab treatment for 2 years was well tolerated and was associated with sustained reductions in exacerbations and progressive reductions in mOCS use.

**Take-home Points**

**Study Question:** This study aimed to assess the real-world benefit of 2 years of mepolizumab treatment in patients with severe asthma.

**Results:** After 2 years of mepolizumab treatment, patients with severe asthma experienced reduced exacerbation rates and maintenance oral corticosteroid use, improved symptom control, and stable lung function; no unexpected safety findings were identified.

**Interpretation:** These data highlight the sustained clinical benefits and consistent safety profile of mepolizumab treatment in a real-world population over 2 years.

Patients with severe asthma require high-dose inhaled corticosteroids plus a second controller and/or oral corticosteroids (OCS) to enable disease control and may even remain uncontrolled despite this therapy.^1^ To achieve disease control and minimize the risk of OCS-related complications,^2^ treatment guidelines recommend adding a biologic to inhaled corticosteroids plus controller therapy in eligible patients with severe asthma based on clinical benefits demonstrated in randomized clinical trials.^3^^,^^4^

Mepolizumab is a humanized, monoclonal anti-IL-5 antibody approved in multiple regions worldwide for the treatment of severe asthma with an eosinophilic phenotype, eosinophilic granulomatosis with polyangiitis, hypereosinophilic syndrome, and chronic rhinosinusitis with nasal polyps.^5^, ^6^, ^7^ Data from international clinical trials and regional real-world studies show that mepolizumab reduces exacerbations and OCS use, while improving symptom control in patients with severe asthma.^8^, ^9^, ^10^, ^11^, ^12^, ^13^, ^14^, ^15^, ^16^, ^17^, ^18^, ^19^, ^20^, ^21^, ^22^, ^23^, ^24^ Additionally, the longer-term real-world benefit of mepolizumab has been studied across a limited number of regional real-world studies^25^, ^26^, ^27^, ^28^; however, equivalent international studies are required.

REALITI-A was a 2-year, international, prospective study assessing the real-world clinical effectiveness of mepolizumab in patients with severe asthma.^29^^,^^30^ After 1 year in REALITI-A, patients demonstrated improved disease control with mepolizumab treatment and a reduced need for maintenance OCS and systemic corticosteroids (SCSs) bursts.^30^ Here, we report the impact of sustained mepolizumab therapy on treatment outcomes in severe asthma across a range of health care systems using data from REALITI-A at 2 years.

## Study Design and Methods

The international, prospective, single-arm, observational REALITI-A cohort study (GSK ID No. 204710) included patients with asthma who were newly prescribed mepolizumab treatment by their physician. Full study methods have been described previously.^30^ The reimbursement criteria for mepolizumab in the participating countries are provided in e-Table 1. Mepolizumab (100 mg subcutaneously [SC]) was first administered on the index date, and data were collected for the 1-year premepolizumab (inclusive of preenrollment [365 days prior to the earliest of index or study enrollment] and variable length run-in period) and 2-year follow-up periods. Data were collected retrospectively for the preenrollment period using medical records, corroborated by patient recall. During the 2-year follow-up period, data were collected contemporaneously using usual standard of care practices at each participating site until the censoring date (defined as the earliest of death, withdrawal, or end of 2-year observational period). Here, we report study outcomes at 2 years after initiation of mepolizumab.

Patients provided informed consent prior to study participation. The study protocol, amendments, informed consent forms, and other information requiring preapproval were reviewed and approved by a national, regional, or independent ethics committee or institutional review board (e-Table 2).

### Patients

Eligible patients were aged ≥ 18 years, had a current clinical diagnosis of asthma with a physician decision to initiate mepolizumab treatment, and had relevant medical records for ≥ 1 year prior to enrollment. Patients were excluded if they had received mepolizumab or had participated in an interventional clinical trial in the year prior to enrollment. Previous use of other biologic medications prior to study enrollment was permitted.

### Outcomes

Prespecified outcomes between the premepolizumab and initial 1-year follow-up periods, including the primary outcome (rate of clinically significant asthma exacerbations [CSEs]), have previously been published.^30^

The prespecified outcomes, assessed during the premepolizumab and follow-up periods unless otherwise specified, included the following: (1) patterns of mepolizumab usage (number of patients discontinuing mepolizumab, reasons for discontinuation, proportion of days covered, medication possession ratio, and number of mepolizumab doses during follow-up); (2) rate of CSEs (defined as a deterioration in symptom control requiring SCSs and/or emergency department [ED] visit/hospital admission); (3) rate of exacerbations requiring an ED visit/hospitalization; (4) rate of exacerbations requiring hospitalization (the proportions of patients with ≥ 50% reduction in CSEs, and those with no CSEs, no exacerbations requiring ED visit/hospitalization, and no exacerbations requiring hospitalization were also assessed); (5) change from baseline (28 days preindex) in daily maintenance OCS, and total OCS use (total OCS use was inclusive of both maintenance OCS and SCS bursts expressed as prednisone-equivalent dose) during the follow-up period among patients using OCS at baseline (proportion of patients who discontinued maintenance OCS completely during the follow-up period and the proportion of patients who had ≥ 50% reduction in maintenance OCS dose vs baseline were also assessed); and (6) change from baseline (nearest value 90 days preindex) in Asthma Control Questionnaire (ACQ)-5 score during the follow-up period. ACQ-5 scores were collected voluntarily at baseline and no more than every 3 months during the follow-up period at usual asthma health care visits.

Other prespecified outcomes included change from baseline (nearest value 90 days preindex) in clinic FEV_1_, peripheral blood eosinophil counts, and fractional exhaled nitric oxide (FeNO) concentration during the follow-up period. Adverse events (AEs) considered related to mepolizumab treatment by the investigator were also reported.

The characteristics of patients who continued/discontinued mepolizumab treatment and FeNO levels by baseline blood eosinophil count (< 300, 300 to < 500, ≥ 500 cells/μL) were also assessed post hoc.

### Statistical Analysis

Power calculations for this study have been previously described.^30^ A treatment policy estimand approach for treatment discontinuation was used, where all data collected in the follow-up period were included regardless of whether patients discontinued mepolizumab treatment. The treated population, used for all effectiveness evaluations, included all enrolled patients who received mepolizumab 100 mg SC at index. The safety population, used for all safety evaluations, included all enrolled patients receiving mepolizumab at any dose.

The rate of exacerbations was analyzed using a generalized estimating equation model assuming a negative binomial distribution, with a covariate of treatment period (premepolizumab and follow-up). Estimated mean variance was corrected for within-patient correlation, and the logarithm of time was used as an offset variable. For the likelihood of no exacerbations, data were modeled using a logistic regression model comparing the premepolizumab and follow-up periods via generalized estimating equation, with a covariate of treatment period (premepolizumab and follow-up).

Median daily maintenance OCS dose and total OCS dose (quartile [Q]1, Q3) was calculated for each 4-week period during the follow-up period. The percentage reduction from baseline in maintenance OCS dose was categorized as follows: 100%, 90% to < 100%, 75% to < 90%, 50% to < 75%, > 0% to < 50%, and no change/increase in OCS dose.

Least squares (LS) mean change (95% CI) from baseline in ACQ-5 score and clinic FEV_1_ and ratio to baseline for blood eosinophil count were analyzed using mixed model repeated measures with covariates of time point, country, baseline OCS therapy (use/no use), and ordinal exacerbation number during the premepolizumab period (0, 1, 2, 3, ≥ 4). Changes in FeNO levels were also summarized where collected at usual asthma health care visits.

One year data reported previously at interim analysis were regenerated as part of this final analysis and reflect all study periods of interest within the final study database.

## Results

### Patient Population and Patterns of Treatment Use

This study was conducted between December 30, 2016, and January 5, 2022. In total, 823 and 822 patients were included in the safety and treated populations, respectively (e-Fig 1). One patient began treatment with mepolizumab 300 mg SC at index (the approved dose for treatment of eosinophilic granulomatosis with polyangiitis) and was not included in the treated population.

At the end of the 2-year follow-up period, 73% of patients had no record of mepolizumab discontinuation (Table 1). The most frequently reported primary reasons for mepolizumab discontinuation were perceived lack of efficacy (n = 77, 9%), switch to another biologic agent (n = 48, 6%), and patient decision (n = 42, 5%). During the 2-year follow-up period, the median proportion of days covered for mepolizumab was 91.0% (Q1, 84.0; Q3, 95.8), with a median medication possession ratio of 92.2% (Q1, 87.7; Q3, 96.6). A median of 24.0 (Q1, 14.0; Q3, 25.0) doses of mepolizumab were received during the follow-up period. The time to discontinuation of mepolizumab is shown in e-Figure 2.

**Table 1:** Patient Treatment Disposition (N = 822)

	Treated Population
No record of mepolizumab discontinuation	599 (73)
Study completed	497 (60)
Withdrawn from study^a^	102 (12)
Mepolizumab discontinued	223 (27)
Follow-up off treatment	32 (4)
Withdrawn from study^b^	191 (23)
Mepolizumab treatment adherence	
Medication possession ratio (n = 812), %	92.2 (87.7, 96.6)
Proportion of days covered (n = 823), %	91.0 (84.0, 95.8)
Mepolizumab doses during follow-up (n = 823), %	24.0 (14.0, 25.0)
Reasons for discontinuation	
Perceived lack of efficacy	77 (9)
Switch to another biologic^c^	48 (6)
Patient decision	42 (5)
Other	20 (2)
AE	18 (2)
Investigator discretion	18 (2)

Values are No. (%) or median (quartile 1, quartile 3). AE = adverse event.

a Without a record of mepolizumab discontinuation.

b Included patients who discontinued mepolizumab and were withdrawn from the study at the same time or who discontinued mepolizumab and were followed up off treatment but were withdrawn from the study prior to month 24.

c Some patients may have switched to another biologic agent due to a perceived lack of efficacy.

Patient baseline demographics and characteristics for the 2-year patient population are shown in Table 2. Baseline patient characteristics were generally similar between those who discontinued (n = 223) vs continued (n = 599) mepolizumab for the entire follow-up period (Table 2); however, those who discontinued vs continued were numerically younger, had a lower blood eosinophil count at baseline, had higher baseline daily maintenance OCS use, and more frequently had previous omalizumab use. Among the treated population, the most common comorbidities were hay fever (n = 405, 49%), chronic sinusitis (n = 331, 40%), nasal polyps (n = 323, 39%), and gastroesophageal reflux disease (n = 310, 38%) (Table 2).

**Table 2:** Patient Demographics and Clinical Characteristics in Patients Who Continued and Discontinued Mepolizumab Treatment During the 2-Year Follow-Up Period

	Total Population (N = 822)	Did Not Discontinue Mepolizumab (n = 599)	Discontinued Mepolizumab (n = 223)
Age, y	54.0 [13.6]	55.2 [12.9]	51.1 [14.9]
Female	521 (63)	375 (63)	146 (65)
BMI, kg/m^2^	(n = 819) 29.0 [7.2]	(n = 598) 28.7 [7.0]	(n = 221) 30.1 [7.8]
Race	n = 821	n = 598	n = 223
Asian or Pacific islander	34 (4)	20 (3)	14 (6)
Black	25 (3)	15 (3)	10 (4)
White	755 (92)	559 (93)	196 (88)
Other	7 (< 1)	4 (< 1)	3 (< 1)
Smoking history	n = 816	n = 595	n = 221
Never smoked	503 (62)	365 (61)	138 (62)
Previous tobacco use	290 (36)	219 (37)	71 (32)
Active tobacco use	23 (3)	11 (2)	12 (5)
Asthma duration, y	(n = 801) 19.7 [15.7]	(n = 587) 19.6 [15.7]	(n = 214) 19.7 [15.6]
Age at asthma diagnosis, y	(n = 801) 34.5 [19.4]	N/A	N/A
Blood eosinophil count, cells/mL,^a^ geo mean [SD log]	(n = 614) 350 [1.25]	(n = 449) 371 [1.24]	(n = 165) 299 [1.28]
Total IgE, KU/L,^a^ geo mean [SD log]	(n = 675) 168.6 [1.59]	(n = 490) 169.8 [1.53]	(n = 185) 165.6 [1.75]
Maintenance OCS	320 (39)	226 (38)	94 (42)
Dose,^b^ mg/d, median (Q1, Q3)	(n = 297) 10.0 (5.0, 14.7)	(n = 209) 8.1 (5.0, 10.2)	(n = 88) 10.7 (7.3, 20.0)
Patients with previous omalizumab treatment	151 (18)	100 (17)	51 (23)
Duration of omalizumab, mo, median (Q1, Q3)	24.0 (6.0, 50.0)	24.0 (9.0, 52.5)	16.0 (5.0, 48.0)
Comorbidities			
Hay fever	405 (49)	302 (50)	103 (46)
Chronic sinusitis	331 (40)	252 (42)	79 (35)
Nasal polyp	323 (39)	247 (41)	76 (34)
Gastroesophageal reflux disease	310 (38)	228 (38)	82 (37)
Drug hypersensitivity	269 (33)	187 (31)	82 (37)

Values are No. (%), mean [SD], or as otherwise indicated. KU, kilounits; NA = not applicable; OCS = oral corticosteroid; Q = quartile.

a The value taken at mepolizumab treatment initiation or the most recent value available within the 90-d period prior to and including mepolizumab treatment initiation.

b Prednisone-equivalent dose in the 28 d prior to and including mepolizumab treatment initiation (baseline); missing baseline maintenance OCS data excluded 23 patients.

### Exacerbation Outcomes

**Figure 1:**
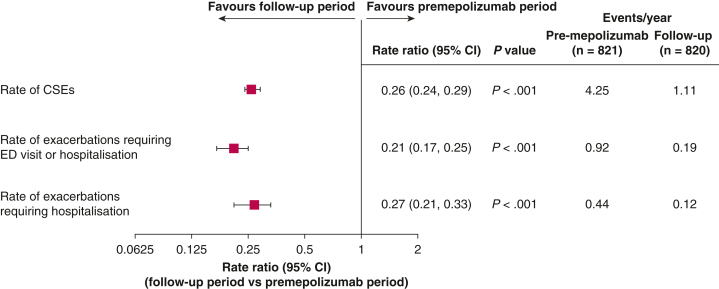
Rate ratios of exacerbations in the 2-y follow-up period vs the premepolizumab period in patients with severe asthma. CSEs were defined as a deterioration in symptom control requiring systemic corticosteroids and/or ED visit/hospital admission. The premepolizumab period consisted of the 365 d prior to enrollment into the study, and the follow-up period included up to 104 wk after mepolizumab treatment initiation and was reported per patient until the earliest of death, study withdrawal, end of the follow-up period, switch to another biologic, or off-label dose of mepolizumab. CSE = clinically significant asthma exacerbation; ED = emergency department.

**Figure 2:**
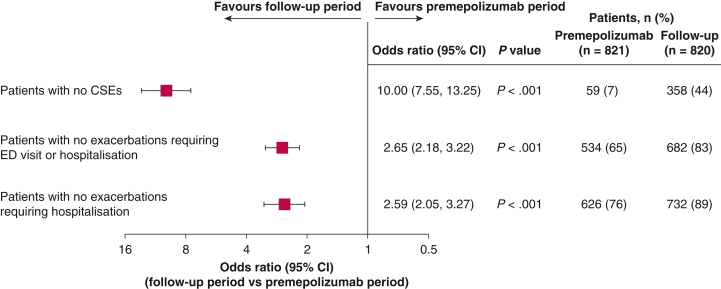
Odds ratios of no exacerbations in the 2-y follow-up period vs the premepolizumab period in patients with severe asthma. The premepolizumab period consisted of the 365 d prior to enrollment into the study, and the follow-up period included up to 104 wk after mepolizumab treatment initiation and was reported per patient until the earliest of death, study withdrawal, end of the follow-up period, switch to another biologic, or off-label dose of mepolizumab. CSEs were defined as a deterioration in symptom control requiring systemic corticosteroid and/or ED visit/hospital admission. CSE = clinically significant asthma exacerbation; ED = emergency department.

The rates of CSEs, exacerbations requiring an ED visit/hospitalization, and exacerbations requiring hospitalization were lower during the 2-year follow-up period vs the premepolizumab period (Fig 1). Among the 819 patients with both premepolizumab and follow-up exacerbation data, 575 (70%) achieved a ≥ 50% reduction in the rate of CSEs. The proportion of patients with no CSEs was higher during the follow-up vs premepolizumab period, as was the proportion of patients with no exacerbations requiring an ED visit/hospitalization or no exacerbations requiring hospitalization (Fig 2). Results were consistent when assessed as a single 2-year period and in 1-year increments (e-Figs 3, 4).

### Maintenance OCS Use

**Figure 3:**
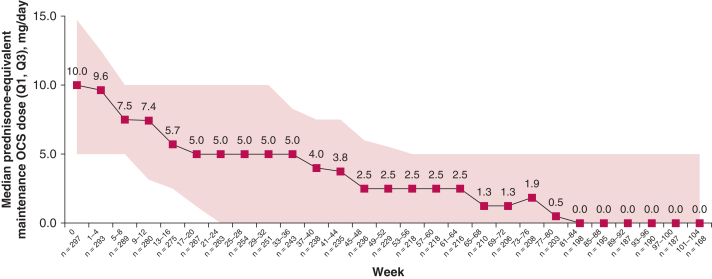
Median maintenance OCS dose over the 2-y follow-up period for patients with severe asthma with baseline maintenance OCS use. Baseline maintenance OCS dose was defined as the average daily dose in the 28 d prior to mepolizumab treatment initiation inclusive of maintenance use only; 320 patients (39%) had baseline maintenance OCS use, and for 23 of these patients, baseline prednisone equivalent maintenance OCS dose could not be calculated. OCS = oral corticosteroid; Q = quartile.

**Figure 4:**
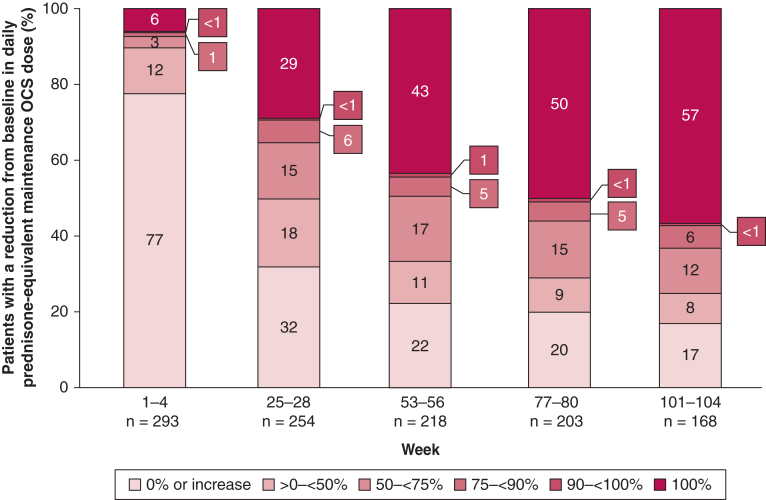
Percentage reduction in daily maintenance OCS dose between baseline and follow-up for patients with severe asthma with baseline maintenance OCS use. Baseline maintenance OCS dose was defined as the average daily dose in the 28 d prior to mepolizumab treatment initiation; 320 patients (39%) had baseline maintenance OCS use, and for 23 of these patients, baseline prednisone equivalent maintenance OCS dose could not be calculated. OCS = oral corticosteroids.

Among the 297 patients with baseline maintenance OCS dose information, median daily prednisone-equivalent maintenance OCS dose decreased from 10.0 mg at week 0 to 0.0 mg at weeks 101 to 104 (168 patients with maintenance OCS dose information at weeks 101 to 104) (Fig 3). Among 308 patients with baseline total OCS dose information (including both maintenance and rescue use), median daily prednisone-equivalent total OCS dose also decreased from 10.0 mg at week 0 to 0.0 mg at weeks 101 to 104 (176 patients with total OCS dose information at weeks 101 to 104) (e-Fig 5). During the follow-up period, the proportion of patients who completely discontinued maintenance OCS use increased from 6% (18 of 293) at weeks 1 to 4 to 43% (94 of 218) at weeks 53 to 56 and 57% (95 of 168) at weeks 101 to 104; conversely, 43% (73 of 168) were unable to reach 0 mg/d at weeks 101 to 104. A total of 75% (126 of 168) had a ≥ 50% reduction in their maintenance OCS dose vs baseline at the weeks 101 to 104 time point (Fig 4), with 83% (139 of 168) having a mean daily maintenance OCS dose of ≤ 5 mg/d. Notably, 29 patients (17%) had no reduction or an increase in maintenance OCS dose at weeks 101 to 104.

### Other Outcomes

Improvements from baseline were seen in ACQ-5 scores, FEV_1_, and blood eosinophil count from the earliest assessed time point, which were then maintained through to the end of study follow-up. Baseline Least squares mean ACQ-5 scores were reduced by 1.30 points at month 6, with a further reduction at month 24 (1.53-point reduction from baseline), well exceeding the minimal clinically important difference of 0.5 points for this questionnaire (Table 3).^31^ FEV_1_ improved by 140 mL from baseline at month 6, with sustained improvements of 142 mL at months 21 to 24 (Table 3). Least squares mean blood eosinophil count decreased from baseline by months 3 to 6, and this reduction was sustained at months 21 to 24. FeNO levels were stable between baseline and months 21 to 24 (Table 3). When stratified by baseline blood eosinophil count, median FeNO level at baseline was higher with increasing blood eosinophil count (cells/μL: < 300 [27.0 parts per billion (ppb)], 300 to < 500 [38.5 ppb], and ≥ 500 [44.8 ppb]) (e-Table 3). Except for the < 300 cells/μL blood eosinophil count subgroup, median FeNO decreased 3 to 6 months after mepolizumab initiation and stabilized throughout the rest of the follow-up period.

**Table 3:** Additional Outcomes Reported Over the 2-Year Follow-Up Period

ACQ-5 Score	Baseline^a^	Month 6	Month 12	Month 18	Month 24
No.	781	338	218	166	194
LS mean (95% CI)	2.87 (2.78–2.96)	1.57 (1.46–1.68)	1.60 (1.47–1.74)	1.52 (1.36–1.67)	1.33 (1.20–1.46)
Change from baseline, LS mean (95% CI)	N/A	−1.30 (−1.42 to −1.18)	−1.26 (−1.41 to −1.11)	−1.35 (−1.51 to −1.19)	−1.53 (−1.67 to −1.40)

Clinic FEV_1_, mL	Baseline^a^	Months 3-6	Months 9-12	Months 15-18	Months 21-24
No.	398	324	257	212	165
LS mean (95% CI)	1,966 (1,890–2,042)	2,106 (2,022–2,191)	2,123 (2,033–2,213)	2,141 (2,056–2,227)	2,108 (2,014–2,202)
Change from baseline, LS mean (95% CI)	N/A	140 (70–210)	157 (81–234)	176 (98–254)	142 (53–231)

Blood Eosinophil Count, cells/μL	Baseline^a^	Months 3-6	Months 9-12	Months 15-18	Months 21-24
No.	614	341	254	173	121
LS mean (95% CI)	350 (320–380)	60 (50–60)	60 (60–70)	60 (50–70)	60 (50–80)
Ratio to baseline, LS mean (95% CI)	N/A	0.16 (0.14–0.19)	0.18 (0.16–0.22)	0.17 (0.14–0.20)	0.18 (0.14–0.23)

FeNO Level, ppb	Baseline^a^	Months 3–6	Months 9–12	Months 15–18	Months 21–24
No.	360	310	210	154	97
Median (Q1, Q3)	39.0 (21.0, 72.0)	33.0 (19.9, 57.5)	30.5 (19.0, 60.0)	34.8 (18.7, 57.0)	32.0 (19.0, 57.3)
No.		236	154	99	63
Median ratio to baseline (Q1, Q3)	N/A	0.87 (0.59, 1.42)	0.91 (0.58, 1.50)	0.86 (0.53, 1.50)	0.83 (0.48, 1.58)

ACQ = Asthma Control Questionnaire; FeNO = fractional exhaled nitric oxide; LS = least squares; N/A = not applicable; ppb = parts per billion; Q = quartile.

a Value taken at mepolizumab treatment initiation or the most recent value available within the 90-d period prior to and including mepolizumab treatment initiation.

### Safety

Investigator-determined treatment-related AEs were experienced by 90 patients (11%) during the follow-up period; serious AEs (SAEs) occurred in 7 patients (< 1%) (Table 4). The most common AE was headache, occurring in 33 patients (4%). One patient had a fatal SAE (diffuse liver malignancy/hepatic cancer), listed as related to mepolizumab treatment by the investigator but the association is uncertain as the patient was also on additional immunosuppressant therapy and monitoring has not to date identified any disproportionate signals for malignancy with mepolizumab.

**Table 4:** AEs and SAEs Related to Mepolizumab Treatment Reported During the 2-Year Follow-Up Period

	Safety Population (N = 823)
Any treatment-related AEs	90 (11)
Leading to discontinuation of mepolizumab	18 (2)
Leading to study withdrawal^a^	11 (1)
Any treatment-related SAEs	7^b^ (< 1)
Fatal	1 (< 1)

Treatment-related AEs and treatment-related SAEs were determined by the investigator. Values are reported as No. (%). AE = adverse event; SAE = serious adverse event.

a All AEs leading to withdrawal from the study also led to discontinuation of treatment with mepolizumab.

b Treatment-related SAEs were bronchospasm (n = 1), pharyngeal swelling (n = 1), pulmonary embolism (n = 1), abdominal pain (n = 1), drug hypersensitivity (n = 1), hepatic cancer (n = 1; fatal), and vitiligo (n = 1).

## Discussion

This large, prospective, international cohort study examined the impact of 2 years of mepolizumab treatment on patients with severe asthma, demonstrating that the previously reported benefits resulting from 1 year of treatment^30^ were sustained at 2 years. Mepolizumab treatment for 2 years in patients with severe asthma was associated with sustained reductions in exacerbations of all severities and a progressive reduction in maintenance OCS use over time. In addition, clinically significant reductions in symptoms and improvements in lung function were observed and the safety data continue to support the favorable real-world safety profile of mepolizumab. This study included a large population from seven countries with varying health care systems and reimbursement criteria^32^, and enrolled patients with a broad range of real-world patient characteristics compared with clinical trials, building on existing data from clinical trials and smaller real-world studies. Furthermore, given the range of clinical benefits seen with mepolizumab in this study, these data provide a baseline for further examination of the concept of clinical remission with biologic treatment, a relatively novel concept and potential goal in the management of severe asthma.^33^

Overall, 83% and 73% of patients had no evidence of mepolizumab discontinuation after 1 and 2 years, respectively (Table 1). The rate of discontinuation from mepolizumab was consistent throughout the 2-year postexposure period; 223 patients (27%) discontinued after 2 years, with 142 (17%) and 81 (10%) discontinuing between 0 and 1 years, and 1 and 2 years, respectively. Patients who discontinued mepolizumab tended to have numerically lower blood eosinophil counts, higher daily maintenance OCS use, and more frequent previous omalizumab use than patients who continued (Table 2). The high adherence to mepolizumab over 2 years (91% proportion of days covered), consistent with the 1-year results,^30^ likely reflects the clinical benefits gained and the monthly administration schedule, and the option for self-administration (Table 1).^5^^,^^6^^,^^34^

During the 2-year follow-up period, the rates of CSEs, exacerbations requiring an ED visit or hospitalization, and exacerbations requiring hospitalization were significantly reduced by 74%, 79%, and 73%, respectively, compared with the premepolizumab period (Fig 1). Additionally, the proportion of patients without an exacerbation increased during the follow-up vs premepolizumab period, across all exacerbation severities (Fig 2). Notably, the odds ratio for not experiencing a CSE during the follow-up vs the premepolizumab period was approximately fourfold higher than the odds ratio for not experiencing a more severe exacerbation (ie, those requiring an ED visit or hospitalization). This difference reflects the statistical power because most (93%) patients enrolled in REALITI-A experienced a CSE during the premepolizumab period, whereas only 24% to 35% had an exacerbation requiring an ED visit or hospitalization during the year prior to the study, meaning 65% to 76% had no ability to show a treatment-related improvement. These global results are consistent with real-world, regional evidence assessing the benefit of mepolizumab over 2 years,^25^, ^26^, ^27^ in addition to shorter-term real-world data,^12^, ^13^, ^14^, ^15^, ^16^, ^17^, ^18^, ^19^ demonstrating the consistency of benefit with mepolizumab across a range of health care systems. They are also broadly consistent with randomized controlled studies of 24 to 52 weeks of mepolizumab treatment, which demonstrated 32% to 58% reductions in clinically significant exacerbations compared with placebo.^8^, ^9^, ^10^ Furthermore, the 2-year REALITI-A study demonstrates that reductions seen at 1 year are maintained with continued treatment for a second year, without loss of effectiveness.

In addition to achieving disease control and avoiding repeated bursts of OCSs for exacerbations, severe asthma management with biologic therapy aims to enable reduction in maintenance OCS dose in those already escalated to this level of therapy.^3^ In REALITI-A, mepolizumab treatment was associated with a progressive reduction in maintenance OCS use over the 2-year follow-up period, with 43% of patients discontinuing maintenance OCSs after 1 year increasing to 57% after 2 years and the median daily maintenance OCS dose reducing by 75% after 1 year and 100% after 2 years (Fig 4).^30^ This dose reduction was as determined between the clinician and the patient, without any specified algorithm. It could be speculated that the pattern of reduction, with plateaus at 5.0 and 2.5 mg (Fig 3), is perhaps consistent with a clinical observation period during reduction, to ensure that there was no rebound deterioration or problems with adrenal suppression. This aligns with the consensus statements of the Delphi Expert Panel strategy, which suggest that tapering should proceed at a slower pace once an agreed threshold has been met and patients should be regularly assessed for adrenal insufficiency.^35^ The progressive reduction in OCS dose over 2 years is consistent with a retrospective report of a 2-year real-world mepolizumab responder analysis in severe asthma.^27^ In this study, maintenance OCS dose was reduced in the first year of mepolizumab treatment (from 10 mg/d at baseline to 5 mg/d), with a further reduction (to 0 mg/d) in the sustained responder population during the second year of treatment.^27^ In REALITI-A, 83% of patients either discontinued OCSs completely or were able to reduce their OCS dose to ≤ 5 mg/d at 2 years. There was no formal assessment of adrenal suppression to evaluate whether those on such lower doses needed OCSs for adrenal replacement rather than asthma. Despite most patients being able to reduce maintenance OCS dose, 17% had no reduction or an increase in maintenance OCS dose during the 2-year follow-up period. Further research is required to determine if patients who are unable to reduce their maintenance OCS use have a disease pathophysiology distinct from eosinophilic inflammation. The number of patients with available data for maintenance OCS use decreased from 320 at baseline to 176 at 2 years; this may suggest that those who discontinued mepolizumab may have done so because of an inability to reduce their maintenance OCS dose.

An improvement was observed in lung function, in addition to a sustained clinically important improvement^31^ in symptoms, and maintained over the 2-year follow-up period; these changes were paralleled by a reduced blood eosinophil count from the first time point postbaseline, sustained until 2 years in accordance with the disease pathology of severe asthma with an eosinophilic phenotype and known mechanism of action of mepolizumab (Table 3).^5^^,^^6^^,^^36^ Previous evidence suggests improved lung function may be an important factor in the decision to continue asthma treatment.^16^^,^^37^ Therefore, improvements in lung function from this study may provide initial evidence of the long-term benefits of mepolizumab for this important guideline-defined treatment goal.^3^

These real-world results show similar improvements in study outcomes and complement previous data from randomized controlled trials (RCTs) (e-Table 4).^8^, ^9^, ^10^ Fewer patients achieved a ≥ 50% reduction in exacerbation in the Steroid Reduction with Mepolizumab Study (SIRIUS) compared with the current REALITI-A study. This difference is likely due to patients enrolled into the SIRIUS study not requiring a prior history of exacerbation events and the use of a protocol-defined OCS tapering schedule, which could have potentially induced additional exacerbations. Additionally, the longer follow-up period of REALITI-A compared with prior RCTs is likely a factor in the larger reductions in OCS dose and improvements in ACQ-5 score observed in the REALITI-A study. Caution is required when comparing the results of REALITI-A with prior RCTs given the lack of a placebo group for direct comparison of outcomes in REALITI-A. Furthermore, there are differences between the patient populations of REALITI-A and mepolizumab RCTs, with REALITI-A including patients with more severe disease (with a greater number of previous exacerbations, and higher blood eosinophil counts), and allowing the enrollment of patients previously excluded from the RCTs (eg, patients with active tobacco use). However, despite these differences, the study results indicate the benefit of mepolizumab treatment beyond the strict boundaries of an RCT.

No unexpected safety findings with mepolizumab treatment were identified (Table 4), and the safety profile was consistent with that shown in previous clinical and real-world studies.^8^, ^9^, ^10^, ^11^, ^12^^,^^17^^,^^18^^,^^20^^,^^21^^,^^23^^,^^26^^,^^30^ Together with the previously performed REALITI-A early initiator analysis in 368 patients at 1 year^29^ and the 1-year analysis of the full study population,^30^ these data highlight the sustained benefit of real-world mepolizumab treatment in patients with severe asthma.

Limitations of the REALITI-A study have previously been discussed^30^ and include the single-arm and open-label study design, which resulted in no comparator or blinding for mepolizumab treatment; however, this is typical of real-world assessments. Data were collected in an observational real-world manner; no protocol required assessments were performed and health care practices across sites differed. Therefore, all patients may not have been followed comprehensively and so data availability for some end points (eg, lung function, patient-reported outcomes) were frequently limited during the follow-up period time points, particularly lung function measurements during the COVID-19 pandemic. However, the use of a treatment policy estimand approach mitigated this limitation. Regarding the impact of the COVID-19 pandemic on outcomes, the previous 1-year analysis of REALITI-A found that although the rate of exacerbations was lower during the pandemic period, a sensitivity analysis found that results were not significantly impacted when patients who were still participating in the study during the pandemic were excluded.^30^ Furthermore, physicians could only allocate one criterion from a single-ordered, multiple-choice selection as the reason for discontinuation of study treatment. Moreover, where lack of efficacy was selected, it was not clear if this was due to physician or patient decision. Additionally, enrolled patients were largely from Western Europe; however, a previous study including patients from other regions suggested that most (84%) patients with severe asthma have an eosinophilic phenotype as per the patients in REALITI-A.^38^ The proportion of patients with no record of mepolizumab treatment discontinuation at the end of the study (73%) also included patients who withdrew from the study with assumed mepolizumab continuation (12%). Finally, there may be bias in the study population based on the increased likelihood of patients who do not respond to treatment discontinuing the study.

## Interpretation

Within 2 years of follow-up after mepolizumab treatment initiation, patients with severe asthma experienced reduced exacerbation rates and maintenance OCS use, paralleled by improved symptom control and stable lung function. These data highlight the sustained, real-world clinical benefits of mepolizumab treatment in this population.

## Funding/Support

This study was funded by GSK [GSK ID Grant 204710]. Editorial support (in the form of writing assistance, including preparation of the draft manuscript under the direction and guidance of the authors, collating and incorporating authors’ comments for each draft, assembling tables and figures, grammatical editing, and referencing) was provided by Katie Crossland, PhD, and Nichola Nolan, MSc, at Fishawack Indicia Ltd, UK, part of Avalere Health, and was funded by GSK.

## Financial/Nonfinancial Disclosures

The authors have reported to *CHEST Pulmonary* the following: C. C. has received research grants and lecture fees from GSK and AstraZeneca. G. W. C. reports research grants and fees from A. Menarini, ALK-Abello, Allergy Therapeutics, AstraZeneca, Boehringer Ingelheim, Chiesi Farmaceutici, Genentech, Guidotti-Malesci, GSK, Hal Allergy, Mylan, Merck, Merck Sharp & Dome, Mundipharma, Novartis, Regeneron, Roche, Sanofi-Aventis, Sanofi-Genzyme, Stallergenes-Greer, UCB Pharma, Uriach Pharma, Valeas, and Vibor-Pharma. M. P. has received lecturing fees and participated in clinical trials with AstraZeneca and GSK; and participated in clinical trials with Chiesi, Oncimmune, Novartis, and Teva. A. S. has received lecturing fees and participated in clinical trials with AstraZeneca, Chiesi, and GSK; and participated in clinical trials with Oncimmune, Novartis, and Teva. M. C. L. has received grants for clinical trials from AstraZeneca, Boehringer Ingelheim, GSK, Gossamer Bio, and Sanofi; and personal fees for participation in advisory boards from AstraZeneca, GSK, and Gossamer Bio. A. V. has served as a consultant to AstraZeneca, Novartis, Sanofi, Boehringer Ingelheim, and Mundipharma; and received lecture fees from Chiesi, Novartis, AstraZeneca, Mundipharma, and GSK. T. C. K. reports personal fees for lecturing and advice from Actelion, AstraZeneca, BerlinChemie, Chiesi, GSK, and Novartis. C. P. has received fees for advisory boards, speaker meetings, and research grants from GSK, AstraZeneca, Chiesi, Novartis, Teva, and ALK-Abello. G. C. has acted as a consultant for AstraZeneca, Genentech, Boehringer Ingelheim, and Teva; attended a speaker’s bureau with AstraZeneca, Genentech, and Circassia; received speaker fees from Amgen, Sanofi-Genzyme, and Regeneron; received research grants from AstraZeneca and institutional grants from AstraZeneca, Genentech, Boehringer Ingelheim, and GSK; has clinical trial funding from Amgen, Sanofi-Genzyme, and Regeneron; and is an advisory board member for Amgen, Sanofi-Genzyme, and Regeneron. G. B. reports consultancy and speaker fees from AstraZeneca, Boehringer Ingelheim, Chiesi, GSK, Novartis, Sanofi, and Teva. R. A.-C., R. G. P., R. W. J., L. D., and P. H. are employed by GSK and hold financial equities in GSK.

## Supplementary Data

e-Online Data

e-Online Data

e-Online Data
