# Supplementary figures and images for: Prospective REALITI-A Study: 2-Year Real-World Benefits of Mepolizumab in Severe Asthma
Source: CHEST Pulm. 2024 Sep 16;3(1):100107. doi: 10.1016/j.chpulm.2024.100107 (PMC13419248; doi:10.1016/j.chpulm.2024.100107)

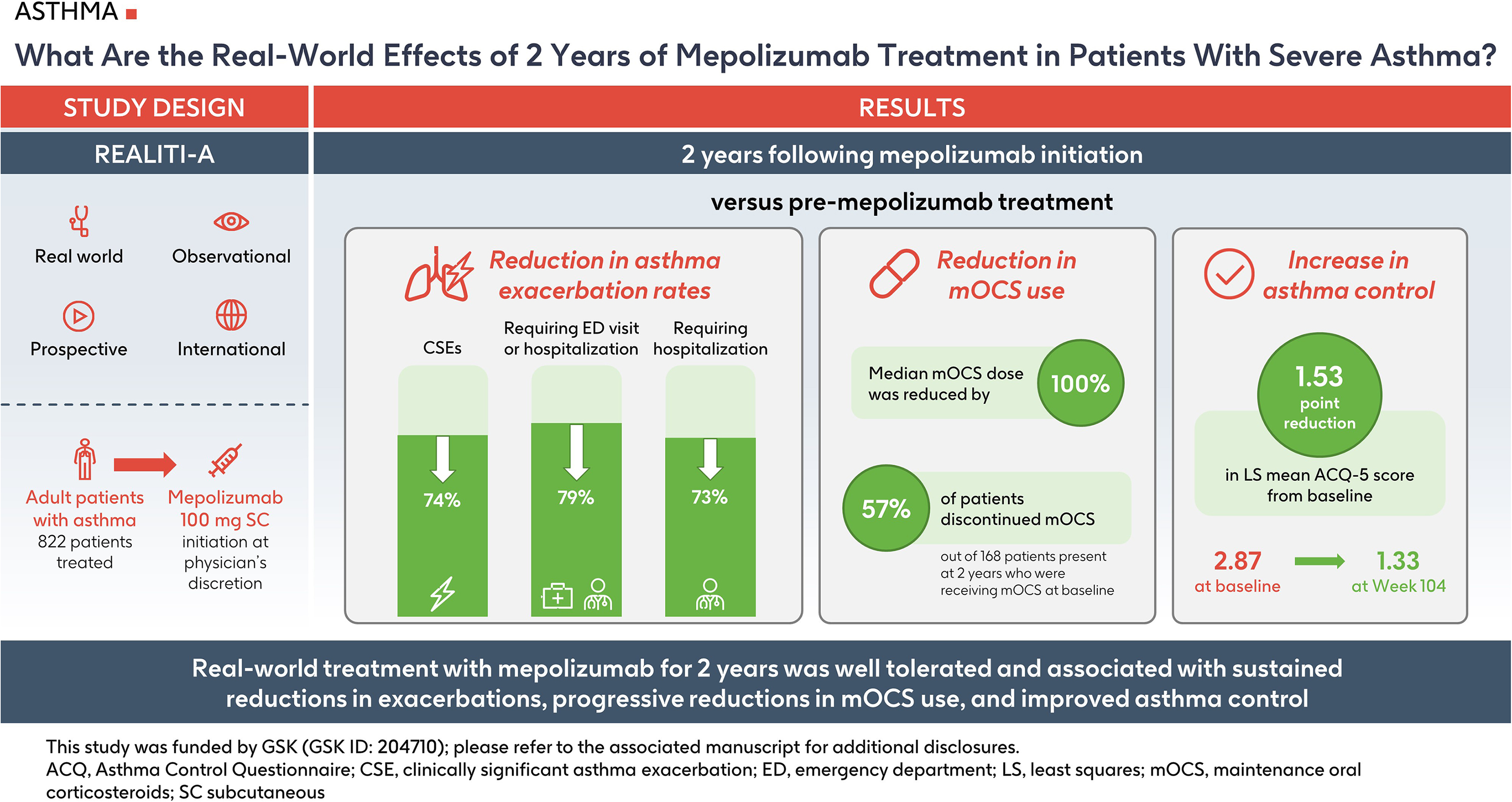

Supplement: e-Online Data [file fx1.jpg]
