# Supplementary material for: Prospective REALITI-A Study: 2-Year Real-World Benefits of Mepolizumab in Severe Asthma
Source: CHEST Pulm. 2024 Sep 16;3(1):100107. doi: 10.1016/j.chpulm.2024.100107 (PMC13419248; doi:10.1016/j.chpulm.2024.100107)
Supplement: e-Online Data [file mmc1.docx]

**Prospective REALITI-A study: 2-year real-world benefits of mepolizumab in severe asthma**

Cristiano Caruso, G Walter Canonica, Manish Patel, Andrew Smith, Mark C Liu, Rafael Alfonso-Cristancho, Robert G Price, Rupert W Jakes, Lydia Demetriou, Antonio Valero, Thomas C Köhler, Charles Pilette, Geoffrey Chupp, Guy Brusselle, Peter Howarth

**Online supplement**

**Figure S1.** Patient disposition.

**Figure S2**. Time to Discontinuation of mepolizumab.

**Figure S3**. Sensitivity analysis of rate ratios of exacerbations in the 2-year follow-up period versus the pre-mepolizumab period in patients with severe asthma.

**Figure S4**. Sensitivity analysis of odds ratios of no exacerbations in the 2-year follow-up period versus the pre-mepolizumab period in patients with severe asthma.

**Figure S5.** Median total OCS dose over the 2-year follow-up period for patients with baseline OCS use.

**Table S1**. Main reimbursement criteria for mepolizumab in the countries participating in the REALITI-A study*.*

**Table S2:** List of independent ethics committees and institutional review boards

**Table S3.** FeNO levels during the follow-up period by baseline blood eosinophil count*.***Table S4.** Comparison of outcomes from randomised-controlled trials and the real-world REALITI-A study.

### **e-Figure S1.** Patient disposition.


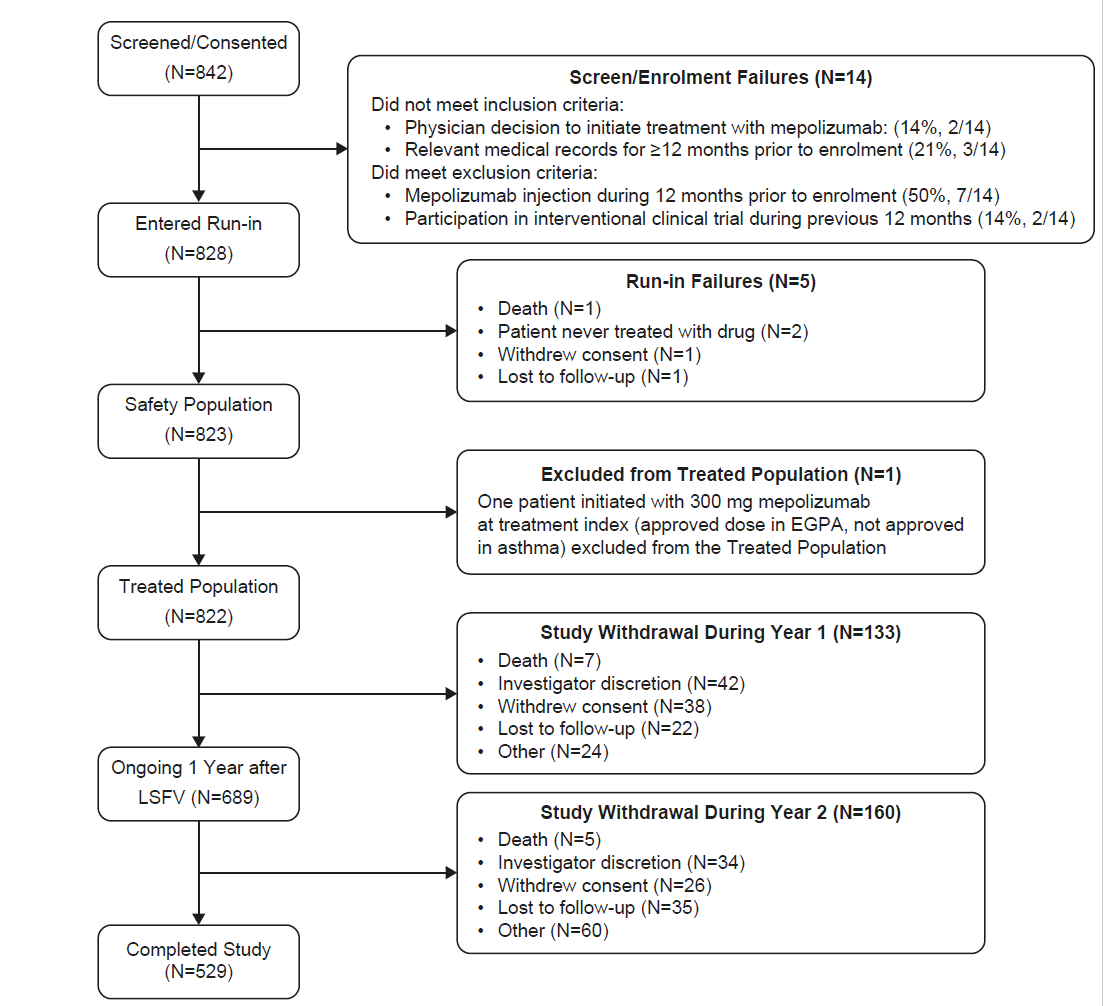

*One patient began treatment with mepolizumab 300 mg SC at index (the approved dose for EGPA) and was not included in the treated population.

EGPA, eosinophilic granulomatosis with polyangiitis; LSFV, last subject first visit; mOCS, maintenance oral corticosteroid; SC, subcutaneous.

### **e-Figure S2.** Time to discontinuation mepolizumab.


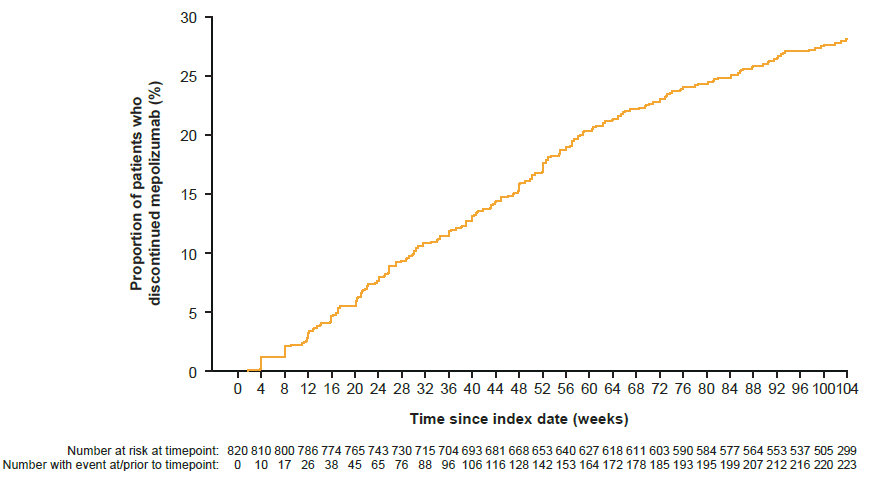


### **e-Figure S3.** Sensitivity analysis of rate ratios of exacerbations in the 2-year follow-up period versus the pre-mepolizumab period in patients with severe asthma.


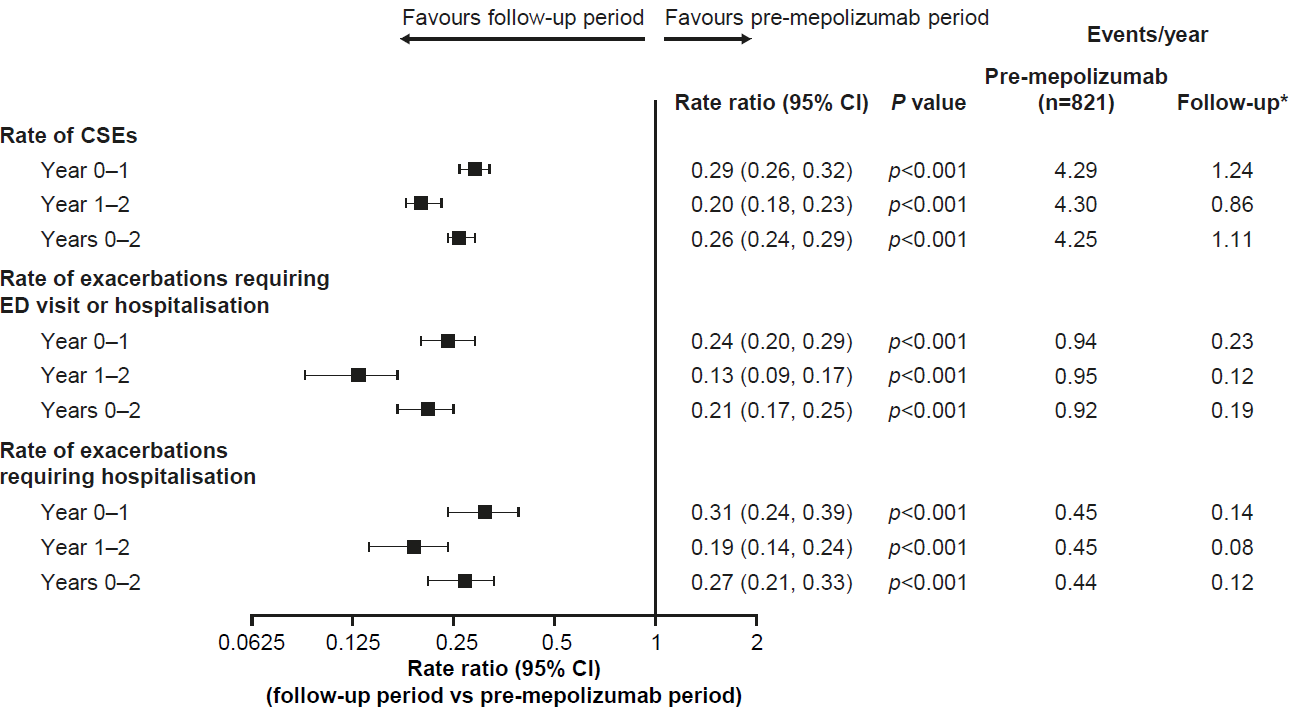


*During follow-up, population sizes were n=820 for Year 0–1, n=670 for Year 1–2, n=820 for Years 0–2. The pre-mepolizumab period consisted of the 365 days prior to enrolment into the study and the follow-up period included up to 104 weeks after mepolizumab treatment initiation and was reported per patient until the earliest of death, study withdrawal, the end of the follow-up period, switch to another biologic, or off-label dose of mepolizumab. CSEs were defined as a deterioration in symptom control requiring SCS and/or ED visit/hospital admission.

The rate of exacerbations was analysed using a generalised estimating equation model assuming a negative binomial distribution, with a covariate of treatment period (pre-mepolizumab and follow-up) using three distinct models (Year 0–1, Years 1–2 and Years 0–2, each vs pre-mepolizumab).

CI, confidence interval; CSE, clinically significant asthma exacerbations; ED, emergency department; SCS, systemic corticosteroid.

### **e-Figure S4.** Sensitivity analysis of odds ratios of no exacerbations in the 2-year follow-up period versus the pre-mepolizumab period in patients with severe asthma.


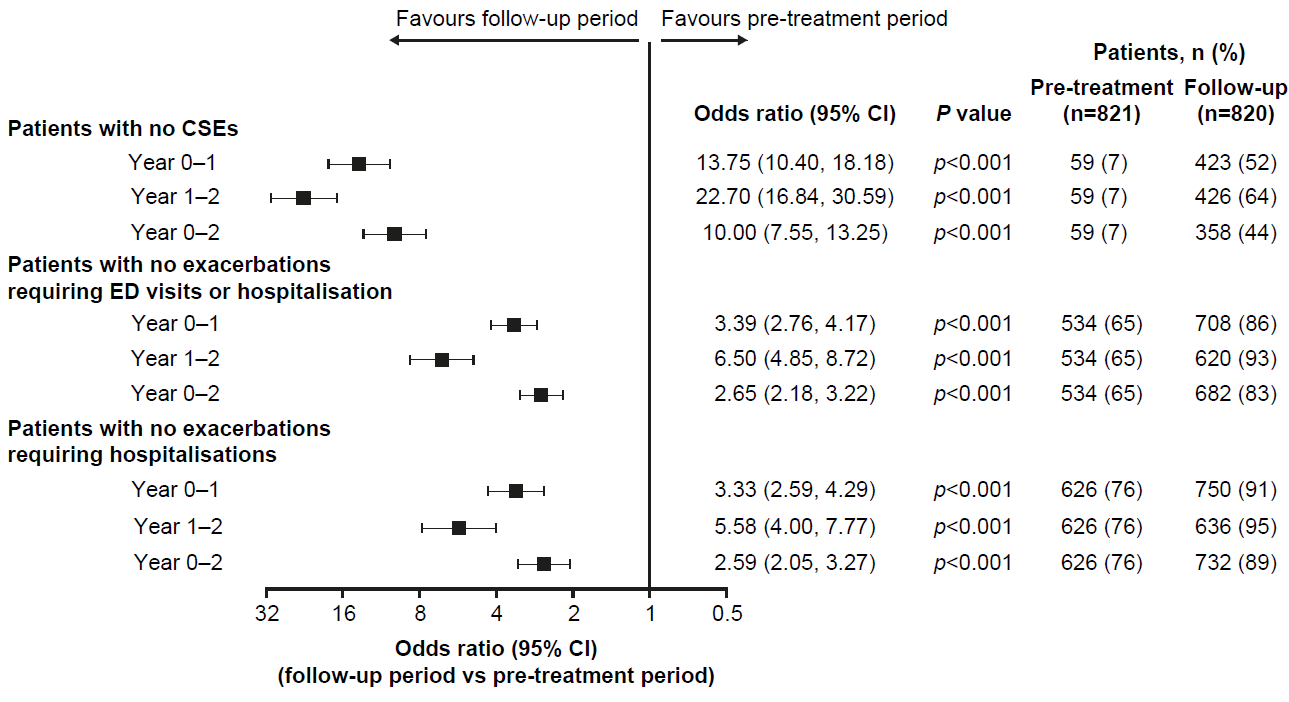


The pre-mepolizumab period consisted of the 365 days prior to enrolment into the study and the follow-up period included up to 104 weeks after mepolizumab treatment initiation and was reported per patient until the earliest of death, study withdrawal, the end of the follow-up period, switch to another biologic, or off-label dose of mepolizumab. CSEs were defined as a deterioration in symptom control requiring SCS and/or ED visit/hospital admission.
The ‘likelihood of no exacerbations’ data were modelled using a logistic regression model comparing the pre-mepolizumab and follow-up periods via generalised estimating equation, with a covariate of treatment period (pre-mepolizumab and follow-up) using three distinct models (Year 0–1, Years 1–2 and Years 0–2 each vs pre-mepolizumab).

CI, confidence interval; CSE, clinically significant asthma exacerbations; ED, emergency department; SCS, systemic corticosteroid.

### **e-Figure S5.** Median total OCS dose over the 2-year follow-up period for patients with baseline OCS use.


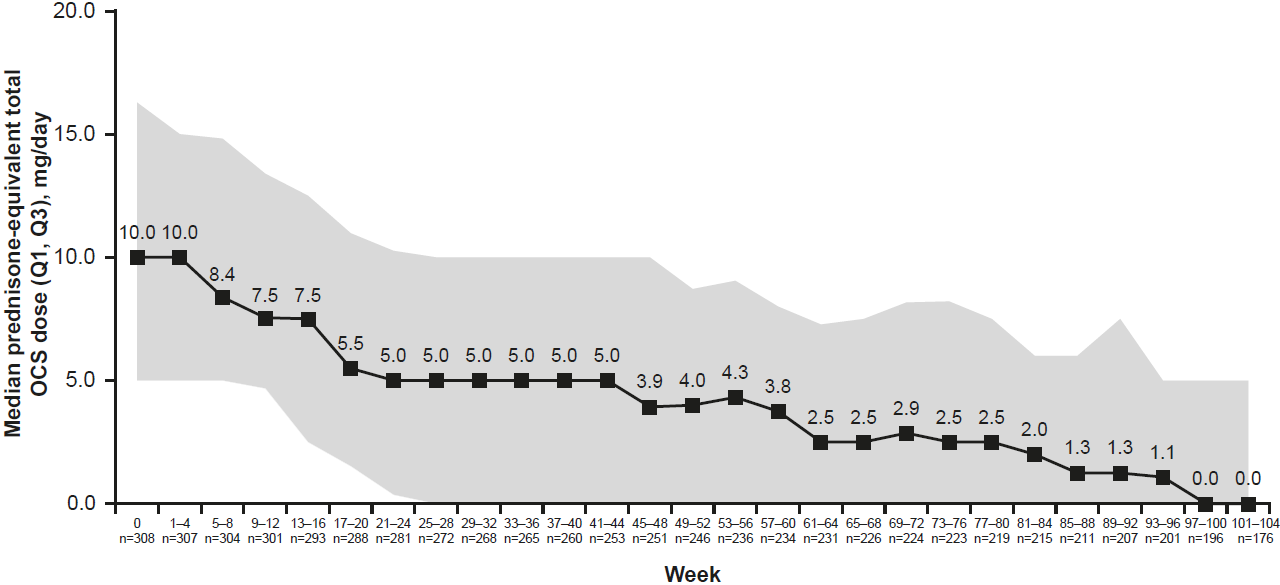


Baseline total OCS dose was defined as the average daily dose in the 28 days prior to mepolizumab treatment initiation inclusive of both maintenance and rescue/exacerbation use; 320 (39%) patients had baseline OCS use and for 12 of these patients, baseline prednisone-equivalent total OCS dose could not be calculated.

OCS, oral corticosteroids; Q, quartile.

### **e-Table S1**. Main reimbursement criteria for mepolizumab in the countries participating in the REALITI-A study.[^38^](#_ENREF_38)

| **Participating country** | **Mepolizumab reimbursement criteria* recommended by national HTA agency** | |
| --- | --- | --- |
|  | **Blood eosinophil count (cells/µL)** | **Exacerbations in the prior 12 months** |
| Belgium (3 centres; n=42) | ≥300 in the past year | ≥2 |
| Canada (7 centres; n=57) | ≥150 at treatment initiation OR ≥300 in the past year | ≥2 |
| Germany (7 centres; n=85) | ≥150 at treatment initiation OR ≥300 in the past year | ≥2 |
| Italy (21 centres; n=244) | ≥150 at treatment initiation AND ≥300 in the past year | ≥2 |
| Spain (11 centres; n=94) | ≥150 at treatment initiation OR ≥300 in the past year | ≥2 |
| UK (11 centres; n=200) | ≥300 in the past year (England, Wales, Northern Ireland); ≥150 at treatment initiation (Scotland) | ≥4 |
| USA (24 centres; n=100) | Varies by individual health plans | Varies by individual health plans |

*Maintenance OCS use and/or level of asthma control may be used as additional criteria in some countries.

HTA, health technology assessment; OCS, oral corticosteroid; UK, United Kingdom; USA, United States of America.

### **e-Table S2.** List of Independent Ethics Committees and Institutional Review Boards

| **Country** | **Investigator no./Center no.** | **Independent Ethics Committees/Institutional Review Boards** |
| --- | --- | --- |
| Belgium | 244317/230826; 087856/230736; 030058/230825; 248636/230829 | Centre Hospitalier Universitaire de Liège, Comité d'Ethique |
| Canada | 005022/230309; 003994/230382; 077977/230384; 007322/238361; 344711/230369; 220890/229775 | Advarra Institutional Review Board |
|  | 173103/230738 | Queen’s University Health Science and Affiliated Teaching Hospitals Research Ethics Board, Office of Research Services |
| Germany | 223054/230468 | Ethik-Kommission der Aerztekammer des Saarlandes |
|  | 392358/230294 | Ethik-Kommission der Landesaerztekammer Baden- Wuerttemberg Medical Association |
|  | 189718/238406; 413648/238363 | Ethik-Kommission der Landesaerztekammer Hessen |
|  | 429964/230293 | Ethik-Kommission der Medizinischen Fakultaet Der Chr. -Albrechts-Universitaet zu Kiel Albert-Ludwigs-University |
|  | 404061/230302 | Ethik-Kommission der Medizinische Fakultaet der Universitaet Duisburg-Essen |
|  | 223342/230292 | Ethik-Kommission der Medizinischen Hochschule Hannover |
|  | 004046/229777 | Ethik-Kommission der Bayerischen Landesaerztekammer Medizinischen Fakultaet Der Chr. -Albrechts-Universitaet zu Kiel Albert-Ludwigs-U |
| Italy | 425130/239567 | Azienda U. Sanitaria Locale Brindisi |
|  | 404827/231354 | Citta della salute e Della scienza di torino Azienda Ospedaliero ordine mauriziano Di Torino ASL Città di Torino |
|  | 423177/238944 | Comitato Etico Azienda Spedali Civili di Brescia |
|  | 232987/231221 | Comitato Etico Catania 1 c/o Azienda Ospedaliero-Universitaria. Policlinico Vittorio Emanuele |
|  | 244886/231293 | Comitato Etico Fondazione Policlinico. A Gemelli – Universita Cattolica del Sacro Cuore |
|  | 344286/232048 | Comitato Etico Indipendente Area 2–Azienda Ospedaliero Universitaria Consorziale Policlinico. Bari |
|  | 012680/231617 | Comitato Etico Indipendente Instituto Clinico Humanitas |
|  | 013464/232668 | Comitato Etico Interprovinciale Area 1 Azienda Ospedaliero-Universitaria, Foggia |
|  | 058109/231266 | Comitato Etico Milano Area 3, Segreteria Scientifico-Amministrativa, c/o ASST Grande Ospedale Metropolitano Niguarda |
|  | 327509/231332 | Comitato Etico per la Sperimentazione dell'Azienda Ospedaliera di Padova |
|  | 185411/232061 | Comitato Etico per Parma, c/o Azienda Ospedaliero Universitaria di Parma |
|  | 344283/232666 | Comitato Etico Province di Chieti e Pescara e Università degli Studi 'Gabriele d'Annunzio' di Chieti-Pescara |
|  | 399915/232689 | Comitato Etico Regione Calabria Sezione. AreaCentro c/o Azienda Ospedaliero-Universitaria. Mater Domini |
|  | 396144/231347 | Comitato Etico Regione Marche - Segreteria Tecnico Scientifica Locale |
|  | 465150/231614 | Comitato Etico Regione Toscana - Area Vasta Centro |
|  | 421201/238611 | Comitato Etico Regione Toscano - Area Vasta Sud Est |
|  | 013662/231019 | Comitato Etico Regione Toscano "Area Vasta Nord Ovest", Segreteria Scientifico-Amministrativa – Azienda Ospedaliero-Universitaria Pisana |
|  | 268686/232667 | Comitato Etico Regionale Dellaliguria (Sezione N.2) |
|  | 013708/231264 | Comitato Etico Sperimentazione Clinica. Provincie Verona e Rovigo, c/o Servizio di Farmacia Azienda Ospedaliero-Universitaria Integrata VR, P.le A. Stefani |
|  | 396016/231304 | Comitato. Etico Universita. Studi Campania Luigi.Vanvitelli – Azienda Ospedaliero-Universitaria Luigi.Vanvitelli – Azienda Ospedaliera di Rilievo Nazionale Ospedali. dei Colli |
|  | 236693/231222 | Fondazione IRCCS Policlinico “san Matteo”, Public Sector Research and Treatment Establishment, Comitato Etico Pavia |
|  | 227899/231615 | Palermo 2 Ethics Committee - c/o Ospedali Riuniti Villa Sofia - Cervello Hospital |
| Spain | 057553/230299; 391218/229987; 011666/230305; 400174/237881; 251900/229778; 403123/238437; 334795/239317; 011183/230301; 233611/230737; 070034/229851; 369812/230308 | CEIC Hospital Clínico San Carlos |
| United Kingdom | 175759/230469;395308/231108; 306061/231185; 376533/230531; 000057/231186; 068733/231220; 227807/231348; 395838/231223; 395292/231277; 396265/230381; 328730/231187 | Scotland A Research Ethics Committee NHS Lothian |
| Unites States of America | 018980/237474; 248809/231021; 132367/238048; 409791/238723; 378643/237948; 022572/231274; 317905/231205; 422088/238738; 147858/240309; 395280/231183; 051145/230373; 425071/239459; 417008/238047; 241507/232184; 176343/238894; 021209/231273; 264578/238248; 011207/231204; 367276/231144; 013337/231018; 393301/230618; 011747/230374; 395256/231079; 418342/238180; 254099/230372; 022259/231131; 268872/230932; 139985/231016; 017249/238808 | Advarra Institutional Review Board |
|  | 013976/237546 | John Hopkins Medicine Institutional Review Board |
|  | 418367/238216; 062590/231957 | Quorum Review Institutional Review Board |
|  | 355925/233964 | Western Institutional Review Board |
|  | 155123/231143 | Yale University Human Investigation Committee |

**e-Table S3.** FeNO levels during the follow-up period by baseline blood eosinophil count.

| **FeNO level (ppb) by baseline* blood eosinophil count** | **Baseline*** | **Months 3–6** | **Months 9–12** | **Months 15–18** | **Months 21–24** |  |
| --- | --- | --- | --- | --- | --- | --- |
| **<300 cells/µL (n=182)** |  |  |  |  |  |  |
| n | 83 | 67 | 48 | 34 | 16 |  |
| Median (Q1, Q3) FeNO level | 27.0 (14.0, 62.0) | 28.7 (18.0, 56.0) | 26.5 (13.6, 46.0) | 26.0 (16.0, 55.0) | 24.0 (17.0, 30.5) |  |
| n |  | 45 | 36 | 16 | 11 |  |
| Median ratio to baseline (Q1, Q3) | N/A | 0.93 (0.71, 1.53) | 0.95 (0.64, 1.35) | 0.95 (0.49, 1.40) | 0.56 (0.43, 1.44) |  |
| **300–<500 cells/µL (n=154)** | | | | | | |
| n | 75 | 69 | 41 | 33 | 15 |  |
| Median (Q1, Q3) FeNO level | 38.5 (19.0, 58.0) | 26.3 (15.2, 46.0) | 28.3 (16.0, 45.3) | 31.0 (16.0, 57.3) | 18.0 (12.0, 78.8) |  |
| n |  | 55 | 30 | 23 | 8 |  |
| Median ratio to baseline (Q1, Q3) | N/A | 0.83 (0.48, 1.14) | 0.90 (0.55, 1.67) | 0.92 (0.72, 1.92) | 0.91 (0.38, 1.51) |  |
| **≥500 cells/µL (n=278)** | | | | | |  |
| n | 134 | 106 | 75 | 56 | 42 |  |
| Median (Q1, Q3) FeNO level | 44.8 (25.0, 77.0) | 36.8 (24.0, 54.3) | 37.5 (23.0, 66.0) | 36.0 (18.5, 51.9) | 37.5 (21.0, 57.3) |  |
| n |  | 86 | 59 | 40 | 31 |  |
| Median ratio to baseline (Q1, Q3) | N/A | 0.82 (0.56, 1.26) | 0.84 (0.52, 1.34) | 0.83 (0.62, 1.41) | 0.97 (0.48, 1.61) |  |

*Value taken at mepolizumab treatment initiation or the most recent value available within the 90-day period prior to and including mepolizumab treatment initiation.

FeNO, fractional exhaled nitric oxide ppb, parts per billion; Q, quartile.

### **e-Table S4.** Comparison of outcomes from randomised-controlled trials and the real-world REALITI-A study.

|  | **MENSA* at Week 32** | **SIRIUS**†  **at Week 24** | **MUSCA**^‡^  **at Week 24** | **REALITI-A**^§^  **at Month 24 (2 years)** |
| --- | --- | --- | --- | --- |
| **≥50% reduction in CSEs vs pre-treatment, n (%)** | n=194 152 (78) | n=69 37 (54) | n=274 225 (82) | n=819 575 (70) |
| **≥50% reduction in OCS dose**^¶^ **vs baseline, n (%)** | N/A | n=69 37 (54) | N/A | n=168 126 (75) |
| **Change from baseline in ACQ-5 score, LS mean (SE)** | n=173 −0.94 (0.07) | n=58 −0.61 (0.13) | n=266 −0.80 (0.06) | n=194 −1.53 (0.07) |
| **Change from baseline in FEV_1_, mL LS mean (SE)** | n=185 183 (31.1) | n=66 111 (55.1) | n=264 176 (26.1) | n=165 142 (44.4) |
| **Ratio to baseline in blood eosinophil count, LS mean (SE Logs)** | n=182 0.14 (0.07) [86% reduction] | n=65 0.21 (0.10) [79% reduction] | n=255 0.19 (0.06) [81% reduction] | n=121 0.18 (0.12) [82% reduction] |

*****MENSA/ MEA115588 (Ortega et al. *N Engl J Med.* 2014;371(13):1198-1207); †SIRIUS / MEA115575 (Bel et al. *N Engl J Med.* 2014;371(13):1189-1197) – steroid sparing trial without prior exacerbation requirement; ^‡^MUSCA / 200862 (Chupp et al. *Lancet Respir Med.* 2017;5(5):390-400.); ^§^Result at Month 24 for ACQ-5 and result at Months 21–24 for clinic FEV_1_ and blood eosinophil counts; ^¶^prednisone equivalent dose

FEV_1_, forced expiratory volume in 1 second; LS, least squares; N/A, not applicable (study did not permit corticosteroid reductions); SE, standard error
