# Supplementary material for: Prospective REALITI-A Study: 2-Year Real-World Benefits of Mepolizumab in Severe Asthma
Source: CHEST Pulm. 2024 Sep 16;3(1):100107. doi: 10.1016/j.chpulm.2024.100107 (PMC13419248; doi:10.1016/j.chpulm.2024.100107)
Supplement: Supplementary file 3 — e-Online Data [file mmc2.docx]

STROBE Statement—checklist of items that should be included in reports of observational studies

|  | Item No. | Recommendation | Page  No. (In “Clean” manuscript) | Relevant text from manuscript |
| --- | --- | --- | --- | --- |
| **Title and abstract** | 1 | (*a*) Indicate the study’s design with a commonly used term in the title or the abstract | Title Page | *“Prospective REALITI-A study: 2-year real-world benefits of mepolizumab in severe asthma”* |
|  |  | (*b*) Provide in the abstract an informative and balanced summary of what was done and what was found | 2–3 | See abstract |
| Introduction | | | |  |
| Background/rationale | 2 | Explain the scientific background and rationale for the investigation being reported | 4 | See introduction for Background  Rationale: “*Data from international clinical trials and regional real-world studies show that mepolizumab reduces exacerbations and OCS use, while improving symptom control in patients with severe asthma.^8-24^ Additionally, the longer-term real-world benefit of mepolizumab has been studied across a limited number of regional real-world studies;^25-28^ however, equivalent international studies are required.*” |
| Objectives | 3 | State specific objectives, including any prespecified hypotheses | 4–5 | “*Here, we report the impact of sustained mepolizumab therapy on treatment outcomes in severe asthma across a range of healthcare systems using data from REALITI-A at 2 years.*” |
| Methods | | | |  |
| Study design | 4 | Present key elements of study design early in the paper | 5 | See ‘Study design an Methods’ section |
| Setting | 5 | Describe the setting, locations, and relevant dates, including periods of recruitment, exposure, follow-up, and data collection | 5 | Full study methods have been described previously (See Reference 30) |
| Participants | 6 | (*a*) *Cohort study*—Give the eligibility criteria, and the sources and methods of selection of participants. Describe methods of follow-up  *Case-control study*—Give the eligibility criteria, and the sources and methods of case ascertainment and control selection. Give the rationale for the choice of cases and controls  *Cross-sectional study*—Give the eligibility criteria, and the sources and methods of selection of participants | 6 | See ‘Patients’ section |
|  |  | (*b*) *Cohort study*—For matched studies, give matching criteria and number of exposed and unexposed  *Case-control study*—For matched studies, give matching criteria and the number of controls per case |  | N/A |
| Variables | 7 | Clearly define all outcomes, exposures, predictors, potential confounders, and effect modifiers. Give diagnostic criteria, if applicable | 6–7 | See ‘Outcomes’ section |
| Data sources/ measurement | 8* | For each variable of interest, give sources of data and details of methods of assessment (measurement). Describe comparability of assessment methods if there is more than one group | 6–7 | See ‘Outcomes’ section |
| Bias | 9 | Describe any efforts to address potential sources of bias |  | N/A |
| Study size | 10 | Explain how the study size was arrived at | 7 | Power calculations for this study have been previously described (see Reference 30). |

Continued on next page

| Quantitative variables | 11 | Explain how quantitative variables were handled in the analyses. If applicable, describe which groupings were chosen and why | 7–9 | See ‘Statistical analysis’ section |
| --- | --- | --- | --- | --- |
| Statistical methods | 12 | (*a*) Describe all statistical methods, including those used to control for confounding | 7–9 | See ‘Statistical analysis’ section |
|  |  | (*b*) Describe any methods used to examine subgroups and interactions |  | N/A |
|  |  | (*c*) Explain how missing data were addressed |  | N/A |
|  |  | (*d*) *Cohort study*—If applicable, explain how loss to follow-up was addressed  *Case-control study*—If applicable, explain how matching of cases and controls was addressed  *Cross-sectional study*—If applicable, describe analytical methods taking account of sampling strategy |  | N/A |
|  |  | (*e*) Describe any sensitivity analyses |  | N/A |
| Results | | | | |
| Participants | 13* | (a) Report numbers of individuals at each stage of study—eg numbers potentially eligible, examined for eligibility, confirmed eligible, included in the study, completing follow-up, and analysed | 9 | “*In total, 823 and 822 patients were included in the safety and treated populations, respectively (****e-Figure S1****)*.” |
|  |  | (b) Give reasons for non-participation at each stage | 9 | “*One patient began treatment with mepolizumab 300 mg SC at index (the approved dose for treatment of EGPA) and was not included in the treated population.*” |
|  |  | (c) Consider use of a flow diagram | Supplementary Material | See **e-Figure S1** |
| Descriptive data | 14* | (a) Give characteristics of study participants (eg demographic, clinical, social) and information on exposures and potential confounders | 9–10 | “*Patient baseline demographics and characteristics for the 2-year patient population are shown in* ***Table 2****. Baseline patient characteristics were generally similar between those who discontinued (n=223) versus continued (n=599) mepolizumab for the entire follow-up period (****Table 2****), although those who discontinued versus continued were numerically younger, had a lower blood eosinophil count at baseline, had higher baseline daily maintenance OCS use, and more frequently had previous omalizumab use. Among the treated population, the most common comorbidities were hay fever (n=405 [49%]), chronic sinusitis (n=331 [40%]), nasal polyps* *(n=323 [39%]) and gastroesophageal reflux disease (n=310 [38%];* ***Table 2****).*” |
|  |  | (b) Indicate number of participants with missing data for each variable of interest | 25–31 | See Table/Figure footnotes |
|  |  | (c) *Cohort study*—Summarise follow-up time (eg, average and total amount) | 25 | See **Table 1** for mepolizumab doses during follow-up |
| Outcome data | 15* | *Cohort study*—Report numbers of outcome events or summary measures over time | 10–12 | See ‘Exacerbation outcomes’, ‘Maintenance OCS use’ ‘ACQ-5 score’ and ‘Other outcomes’ in the Results section |
|  |  | *Case-control study—*Report numbers in each exposure category, or summary measures of exposure |  | N/A |
|  |  | *Cross-sectional study—*Report numbers of outcome events or summary measures |  | N/A |
| Main results | 16 | (*a*) Give unadjusted estimates and, if applicable, confounder-adjusted estimates and their precision (eg, 95% confidence interval). Make clear which confounders were adjusted for and why they were included | 10–12, 28, 30–31 | See ‘Exacerbation outcomes’, ‘Maintenance OCS use’ ‘ACQ-5 score’ and ‘Other outcomes’ in the Results section and Figures 1–4 and Table 3 |
|  |  | (*b*) Report category boundaries when continuous variables were categorized |  | N/A |
|  |  | (*c*) If relevant, consider translating estimates of relative risk into absolute risk for a meaningful time period |  | N/A |

Continued on next page

| Other analyses | 17 | Report other analyses done—eg analyses of subgroups and interactions, and sensitivity analyses |  | N/A |
| --- | --- | --- | --- | --- |
| Discussion | | | | |
| Key results | 18 | Summarise key results with reference to study objectives | 12 | “*This large, prospective, international cohort study examined the impact of 2 years of mepolizumab treatment on patients with severe asthma, demonstrating that the previously reported benefits resulting from 1 year of treatment^30^ were sustained at 2 years. Mepolizumab treatment for 2 years in patients with severe asthma was associated with sustained reductions in exacerbations of all severities and a progressive reduction in maintenance OCS use over time. In addition, clinically significant reductions in symptoms and improvements in lung function were observed and the safety data continue to support the favourable real-world safety profile of mepolizumab.*” |
| Limitations | 19 | Discuss limitations of the study, taking into account sources of potential bias or imprecision. Discuss both direction and magnitude of any potential bias | 17 | See paragraph beginning ‘Limitations of the REALITI-A study…’ |
| Interpretation | 20 | Give a cautious overall interpretation of results considering objectives, limitations, multiplicity of analyses, results from similar studies, and other relevant evidence | 18–19 | “*Within 2 years of follow-up after mepolizumab treatment initiation, patients with severe asthma experienced reduced exacerbation rates and maintenance OCS use, paralleled by improved symptom control and stable lung function. These data highlight the sustained, real-world clinical benefits of mepolizumab treatment in this population*.” |
| Generalisability | 21 | Discuss the generalisability (external validity) of the study results | 14 and 17 | Page 14: “*These global results are consistent with real-world, regional evidence assessing the benefit of mepolizumab over 2 years,^25-27^ in addition to shorter-term real-world data,^12-19^ demonstrating the consistency of benefit with mepolizumab across a range of healthcare systems. They are also broadly consistent with randomised controlled studies of 24–52 weeks of mepolizumab treatment, which demonstrated 32–58% reductions in clinically significant exacerbations compared with placebo.^8-10^ Furthermore, the 2-year REALITI-A study demonstrates that reductions seen at 1 year are maintained with continued treatment for a second year, without loss of effectiveness.*”  Page 17: “*No unexpected safety findings with mepolizumab treatment were identified (Table 4), and the safety profile was consistent with that shown in previous clinical and real-world studies.^8-12,17,18,20,21,23,26,30^ Together with the previously performed REALITI-A early initiator analysis in 368 patients at 1 year^29^ and the 1-year analysis of the full study population,^30^ these data highlight the sustained benefit of real-world mepolizumab treatment in patients with severe asthma.^”^* |
| Other information | |  | | |
| Funding | 22 | Give the source of funding and the role of the funders for the present study and, if applicable, for the original study on which the present article is based | Title page | “*This study was funded by GSK (GSK ID: 204710).*” |

*Give information separately for cases and controls in case-control studies and, if applicable, for exposed and unexposed groups in cohort and cross-sectional studies.

**Note:** An Explanation and Elaboration article discusses each checklist item and gives methodological background and published examples of transparent reporting. The STROBE checklist is best used in conjunction with this article (freely available on the Web sites of PLoS Medicine at http://www.plosmedicine.org/, Annals of Internal Medicine at http://www.annals.org/, and Epidemiology at http://www.epidem.com/). Information on the STROBE Initiative is available at www.strobe-statement.org.
